# Genomic insights into genetic diversity and local adaptation of a dominant desert steppe feather grass, *Stipa breviflora* Griseb.

**DOI:** 10.3389/fpls.2023.1170075

**Published:** 2023-05-17

**Authors:** Dongqing Yan, Jiamei Liu, Yanyan Fan, Zhi Lian, Zhenhua Dang, Jianming Niu

**Affiliations:** ^1^School of Ecology and Environment, Inner Mongolia University, Hohhot, China; ^2^Ministry of Education Key Laboratory of Ecology and Resource Use of the Mongolian Plateau, Ministry of Science and Technology of China, Hohhot, China; ^3^Inner Mongolia Key Laboratory of Grassland Ecology and the Candidate State Key Laboratory of Ministry of Science and Technology, Ministry of Science and Technology of Inner Mongolia Autonomous Region, Hohhot, China

**Keywords:** *Stipa breviflora*, steppe, population differentiation, climatic factors, RPK2, CPRF1

## Abstract

Investigating the genetic mechanisms of local adaptation is critical to understanding how species adapt to heterogeneous environments. In the present study, we analyzed restriction site-associated DNA sequencing (RADseq) data in order to explore genetic diversity, genetic structure, genetic differentiation, and local adaptation of *Stipa breviflora*. In total, 135 individual plants were sequenced and 25,786 polymorphic loci were obtained. We found low genetic diversity (*He* = 0.1284) within populations of *S. breviflora*. Four genetic clusters were identified along its distribution range. The Mantel test, partial Mantel test, and multiple matrix regression with randomization (MMRR) indicate that population differentiation was caused by both geographic distance and environmental factors. Through the *F_ST_* outlier test and environmental association analysis (EAA), 113 candidate loci were identified as putatively adaptive loci. *RPK2* and CPRF1, which are associated with meristem maintenance and light responsiveness, respectively, were annotated. To explore the effects of climatic factors on genetic differentiation and local adaptation of *S. breviflora*, gradient forest (GF) analysis was applied to 25,786 single nucleotide polymorphisms (SNPs) and 113 candidate loci, respectively. The results showed that both temperature and precipitation affected the genetic differentiation of *S. breviflora*, and precipitation was strongly related to local adaptation. Our study provides a theoretical basis for understanding the local adaptation of *S. breviflora*.

## 1 Introduction

Landscape genomics investigates and quantifies the effects of environmental heterogeneity on geographic patterns of genetic variation in natural populations, providing critical insights into the local adaptation of species (Joost et al., 2007; Balkenhol et al., 2017; Li et al., 2017). Environmental factors vary spatially, causing plant populations to evolve different advantageous traits to survive under local stresses (Kawecki and Ebert, 2004; Savolainen et al., 2013). Diverse selection pressures may lead to genetic variation and differentiation of species on a genome-wide scale (Rellstab et al., 2015). Such genetic variation and differentiation along environmental gradients can be indicative of local adaptation (Zhang et al., 2020). In past decades, many studies have documented local adaptation to different environmental factors, such as temperature (Zheng et al., 2011; Körner, 2016; Aguirre-Liguori et al., 2021), soil characteristics (Guerrero et al., 2018), and even atmospheric gases (Watson-Lazowski et al., 2016), across many species, including *Quercus rugosa* (Gugger et al., 2021), *Arabidopsis thaliana* (Lasky et al., 2014), and *Pterocarya stenoptera* (Li et al., 2018). Revealing the molecular basis of the genetic variation caused by heterogeneous environments helps to understand how populations evolve owing to local adaptation.

Investigating how genomic variations contribute to local adaptation and identifying selective forces is still challenging for species with limited genomic resources (Mayol et al., 2020). Nonetheless, the development of genome-scale genotyping approaches, such as restriction site-associated DNA sequencing (RADseq), has made it possible to collect thousands to millions of single nucleotide polymorphisms (SNPs) for non-model species (Savolainen et al., 2013; Sork et al., 2013). Abundant genomic data coupled with effective loci-identifying methods have promoted an understanding of local adaptation. The methods used to identify the loci underlying local adaptation are grouped into two categories: differentiation-based outlier tests (*F_ST_* outlier tests) and environmental association analysis (EAA) (Schoville et al., 2012). *F_ST_* outlier tests are used to detect loci potentially under selection, which exhibit significantly higher values of genetic differentiation (F_ST_) than expected under neutrality (Narum and Hess, 2011). However, this approach may detect some false positive loci associated with evolutionary processes (i.e., genetic drift, population history, and gene flow) other than local adaptation (Aguirre-Liguori et al., 2021). Therefore, EAA, an approach that separates a subset of SNPs that have exceptional environmental associations from the background associations generated by neutral processes, is usually combined with *F_ST_* outlier tests to minimize false positives (Lotterhos and Whitlock, 2015; Li et al., 2017).

The steppe zone is a huge area in temperate Eurasia where different grasslands, dominated by various *Stipa* species, form the main type of vegetation (Pfadenhauer and Klötzli, 2020; Sergeev, 2021). Recent studies have shed light on the demographic history (Vintsek et al., 2022), phylogeny (Krawczyk et al., 2022), hybridization, and introgression events (Baiakhmetov et al., 2020; Baiakhmetov et al., 2021) of some *Stipa* species in Central Asia, advancing our understanding of this genus from different perspectives. Desert steppe, an important steppe formation of the Eurasian steppe, is the ecotone between grassland and desert (Chen et al., 2020). Compared with other grassland types, the desert steppe has far less vegetation, which is susceptible to climate change and anthropogenic disturbances (Zhao et al., 2002; Angerer et al., 2008). *Stipa breviflora* Griseb., as one of the dominant species in the desert steppe, is an important foraging resource because of its palatability, rich nutrient content, early greening, and resistance to grazing and drought (Ren et al., 2017; Yan et al., 2020). Moreover, *S. breviflora* has attracted attention for its potential use for water and soil conservation and for desertification control (Wang et al., 2018; Chen et al., 2020). However, this kind of desert steppe has undergone degradation succession, and the role of *S. breviflora* in constructing communities is likely to change as a consequence of global warming (Zhang et al., 2014; Wang et al., 2015; Wu et al., 2020; Lv et al., 2021).

The distribution of *S. breviflora* covers a large temperature and precipitation range, from the cold and dry climate of the Qinghai-Tibetan Plateau (QTP) to the relatively warm and wet Loess Plateau. However, how this species adapts to these highly heterogeneous habitats, both geographically and ecologically, is still unknown. To uncover the genomic basis of genetic variation and local adaptation of *S. breviflora*, we sampled 135 individual plants belonging to 27 populations from its distribution area. We generated two datasets: all-SNP dataset that derived from the original RADseq, and the outlier dataset that derived from the all-SNP dataset and represents the loci under selection. We generated two datasets: all-SNP dataset that derived from the original RADseq, and the outlier dataset that derived from the all-SNP dataset and represents the loci under selection. Our study will provide information on the inter-relationship existing between heterogeneous environments and genetic variability, which will deepen our understanding of the local adaptation of *S. breviflora*.

## 2 Materials and methods

### 2.1 Study species and sampling

*Stipa breviflora* is a wind-pollinated and selfing facultatively perennial grass (Wan et al., 1997) that is widely distributed across a continuous zone that stretches from the southwest of the Loess Plateau, across the Yinshan Mountains, to the south of the Mongolian Plateau (Zhang et al., 2012). It also dominates the desert steppe, within altitude zones that differ in terms of temperature, precipitation, and soil attributes, in mountains located in the Xinjiang region and the QTP (Zhang et al., 2012; Lv and Zhou, 2018) The plant regreens in early April and sets seeds from May to July. The seeds are characterized by a short plumose awn and spinulose lemma apex that allows for wind or zoochorous dispersal (Ye et al., 2020). It can also be propagated clonally *via* tillering. With its strong ecological adaptability, *S. breviflora* can be codominant with other *Stipa* species such as *Stipa bungeana* in warm–temperate steppes and *Stipa krylovii* in typical steppes (Lu and Wu, 1996).

**Figure 1:**
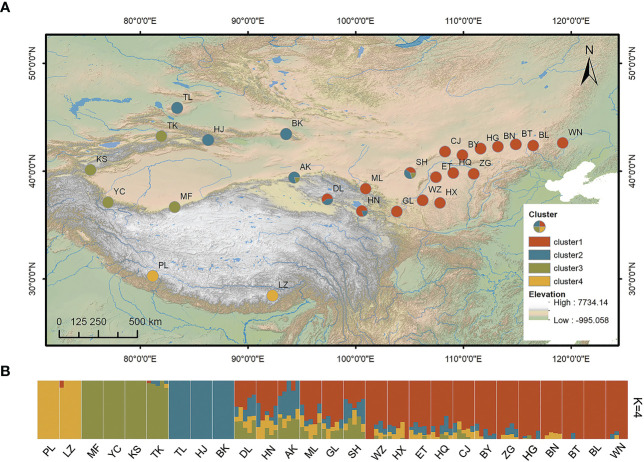
**(A)** Geographical locations of the 27 sampled populations of *Stipa breviflora* and **(B)** genetic structure for *K* = 4 based on admixture analysis. Abbreviations in both **(A, B)**, such as PL and LZ, represent the sampling sites in the present study. K, number of clusters.

The fresh leaves of 135 *S. breviflora* samples were collected from 27 localities throughout the species’ distribution area (**Figure 1A**; **Table 1**). From each population, five individuals were sampled at least 10 m apart to avoid sampling ramets of the same genet. Leaf materials were stored in liquid nitrogen in the field and frozen at –80°C in the laboratory. No specific permits were required for *S. breviflora* sampling and all samples were collected following government regulations.

**Table 1:** Population locations and summary of genetic statistics of *S. breviflora*.

Cluster	Population	Longitude (°)	Latitude (°)	Elevation (m)	n	π	Ho	He	Fis
Cluster 1	WN	119.2	42.6	576.1	5	0.0172	0.0955	0.0839	0.1860
	BL	115.4	42.4	1287.7	5	0.0163	0.1713	0.1534	0.3523
	BT	114.9	42.5	1236.9	5	0.0131	0.1291	0.1156	0.2754
	BN	112.1	42.3	1304.9	5	0.0826	0.1755	0.1564	0.2059
	HG	111.6	42.1	1283.9	5	0.0473	0.1890	0.1686	0.3175
	ZG	111.0	39.8	1160.2	5	0.0268	0.1751	0.1556	0.3315
	BY	108.9	41.5	1330.4	5	0.0575	0.1868	0.1664	0.2863
	CJ	108.3	41.8	1378.2	5	0.0145	0.1983	0.1768	0.4188
	HQ	108.6	39.8	1342.5	5	0.0149	0.1693	0.1571	0.3524
	ET	107.4	39.5	1386.0	5	0.0253	0.1831	0.1635	0.3572
	HX	106.9	37.1	1554.8	5	0.0192	0.2059	0.1827	0.4175
	WZ	106.2	37.3	1687.5	5	0.0230	0.1542	0.1359	0.2741
	SH	105.1	39.8	1461.6	5	0.0117	0.1725	0.1536	0.3569
	GL	103.8	36.3	1784.1	5	0.0964	0.1951	0.1725	0.2135
	ML	100.9	38.4	2440.9	5	0.0227	0.2068	0.1844	0.4133
	HN	100.6	36.3	2956.3	5	0.1146	0.2180	0.1919	0.2299
	DL	97.4	37.4	3049.5	5	0.0175	0.1872	0.1656	0.3706
Cluster 2	AK	94.3	39.4	2623.4	5	0.0218	0.1934	0.1725	0.3848
	BK	93.5	43.5	1980.1	5	0.0126	0.0775	0.0694	0.1515
	HJ	86.3	42.9	2141.2	5	0.0260	0.0676	0.0603	0.0775
	TL	83.4	45.9	1208.4	5	0.0121	0.0778	0.0697	0.1445
Cluster 3	TK	82.0	43.2	1113.6	5	0.0146	0.0932	0.0831	0.1740
	KS	75.4	40.1	2583.2	5	0.0146	0.0931	0.0825	0.1734
	YC	77.0	37.1	2629.5	5	0.0150	0.1215	0.1076	0.2328
	MF	83.2	36.7	2485.1	5	0.0167	0.0936	0.0828	0.1807
Cluster 4	LZ	92.3	28.5	3996.5	5	0.0223	0.0535	0.0472	0.0823
	PL	81.2	30.3	3863.5	5	0.0131	0.0076	0.0068	–0.0099
	mean				5	0.0292	0.1441	0.1284	0.2574

n, number of individuals sampled; *π*, nucleotide diversity; *Ho*, observed heterozygosity; *He*, expected heterozygosity; *Fis*, inbreeding coefficient.

### 2.2 Library construction, sequencing, and data processing

DNA extraction was performed with the Tiangen Plant DNA Extraction Kit DP305 (Tiangen, Beijing, China) in accordance with the manufacturer’s protocol. Extracted DNA was quantified by NanoDrop 2000 UV–Vis spectrophotometers (ThermoFisher Scientific, Waltham, MA, USA). Restriction site-associated DNA (RAD) libraries were prepared and sequenced for each DNA sample by Beijing Genomics Institute (BGI; Shenzhen, China) using the restriction enzyme *Eco*RI and sample-specific barcodes. Samples in the libraries were pooled and sequenced on an Illumina Hiseq X10 to generate 146-bp paired-end reads.

Standard quality control (QC) pipelines (BGI, Shenzhen, China) were used to process the raw sequencing data. Raw reads from the same library were demultiplexed according to index barcodes and reads containing adaptors were removed. Reads with more than 40% low-quality bases (phred scores < 20) and reads with more than 10% “Ns” were discarded using SOAPnuke v1.5.6 (Chen et al., 2018). STACKS v1.48 (Catchen et al., 2013) was used to assemble the clean reads into *de novo* loci, and to call the SNPs using the “denovo_map.pl” module with the following settings: minimum number of reads to create a stack, m = 2; maximum distance allowed between stacks, M = 2; maximum number of mismatches allowed between loci, n = 3; minimum number of populations a locus must be present in, p = 20; minimum percentage of individuals in a population required to process a locus for that population, r = 0.8; minimum minor allele frequency, min-maf = 0.05. In addition, data analysis was restricted to the first SNP per RAD locus, to reduce the impact of linkage disequilibrium (-write_single_snp). The filtered dataset (all-SNP dataset) of 25,786 SNPs was exported in vcf format using the “-vcf” option.

### 2.3 Population genetic diversity, structure, and differentiation

Nucleotide diversity (π), observed heterozygosity (*Ho*), expected heterozygosity (*He*), and inbreeding coefficient (*Fis*) were calculated using the “populations” module in STACKS v1.48 (Catchen et al., 2013). Genetic structure analysis and principal component analysis (PCA) were employed in this study. The population structure was investigated using ADMIXTURE v1.3.0 (Alexander et al., 2009). This program adopts an unsupervised approach to calculate a matrix of ancestry coefficients that are the proportions of an individual genome belonging to different ancestral populations. The input file was converted to “plink” format using VCFtools v0.1.13 (–plink) (Danecek et al., 2011) and to “ped” format using plink v2.0 (Chang et al., 2015). We ran ADMIXTURE with *K* ranging from 1 to 27 and repeated the process 10 times for each *K* (number of clusters) with different random seeds. The most probable number of clusters was inferred by the lowest cross-validation error. We also conducted PCA in plink v2.0 (Chang et al., 2015) to assess the genetic variance. Pairwise population differentiation (*F_ST_*) was computed using the R package “hierfstat” (Goudet, 2005). Analysis of molecular variance (AMOVA) in Arlequin v3.5.2 (Excoffier and Lischer, 2010) was used to quantify the genomic variance between individuals, within and among sample groups, with significance tests based on 10,000 permutations.

### 2.4 Isolation by distance or environment

Environmental data (19 climatic variables for 1970-2000 with a spatial resolution of 1 km) were downloaded from WorldClim (http://www.worldclim.org) (Fick and Hijmans, 2017). PCA was applied to eliminate inter-correlations of these environmental variables and to extract independent climatic gradients. Both environmental and geographical distances were calculated by PASSaGE v2 (Rosenberg and Anderson, 2011). Genetic distances were estimated using the formula *F_ST_/*(1 – *F_ST_*). Mantel and partial Mantel tests were performed using the “vegan” package (Dixon, 2003). Multiple matrix regression with randomization (MMRR), an approach for quantifying geographical and ecological isolation, was implemented with 10,000 permutations in R with the MMRR script (Wang, 2013).

### 2.5 Outlier detection and functional annotation

We used both the *F_ST_* outlier test and EAA to detect outliers. Firstly, Bayescan v2.1 (Foll and Gaggiotti, 2008) was used to detect the loci (under positive or balancing selection) with *F_ST_* that deviated from expectations under a neutral model of selected. Bayescan v2.1 (Foll and Gaggiotti, 2008) was run with 20 pilot runs of 5,000 iterations followed by 100,000 iterations and an additional burn-in of 50,000 iterations. Loci with a false discovery rate (FDR) <0.05 were considered to be outliers. Secondly, to perform EAA, latent factor mixed modeling (LFMM) analysis and 19 bioclimatic factors were used to identify outliers in the LEA package with the “lfmm2” function (Frichot and François, 2015). We set a significance threshold of FDR-adjusted *p* < 0.01 to select loci under natural selection. The intersection of the loci obtained by both Bayescan and LFMM analysis constituted the outlier dataset. To annotate the function of identified outliers, their sequences were mapped against the transcriptome sequences of *S. breviflora* (non-published data) using the program BLASTN (Altschul et al., 1990) with an *E*-value cut-off of 10^–5^, as there is no available genome data of *S. breviflora*. Then, the Kyoto Encyclopedia of Genes and Genomes (KEGG, https://www.kegg.jp/) database and Gene Ontology (GO, http://geneontology.org/) database were used for BLAST search and annotation.

### 2.6 Determination of environmental impacts on genetic variation and local adaptation of *S. breviflora*

Gradient forest (GF) is a non-parametric, machine learning, regression tree approach that uses SNP allele frequencies (as response variables) and climatic data (as predictors) to identify environmental gradients that are associated with genetic variation and also to determine allele frequency turnover along that gradient. We applied gradient forest analysis to the all-SNP dataset and the outlier dataset, using gradientForest_0.1–18 (https://r-forge.r-project.org/R/?group_id=973). R packages “raster” (Hijmans et al., 2015) and “rgdal” (Hijmans et al., 2015) were used to extract 19 climatic variables at each sampling location. After removing variables with a Pearson’s *R^2^* > 0.9, 12 climatic variables were retained to build the final GF model with the default parameters (**Table S1**). Then, The statistical significance of the most important environmental factor generated by GF analysis was then assessed using analysis of variance (ANOVA).

## 3 Results

### 3.1 Sequencing data

A total of 374.48 Gb of sequencing data were generated from 135 individuals of *S. breviflora*. Over 2,546 million reads passed initial quality controls. The mean, minimum, and maximum number of sequencing data for each individual were 18.86 million, 11.38 million, and 25.51 million, respectively (**Table S2**). By assembling and filtering in STACKS v1.48, the final dataset containing 25,786 polymorphic loci was retained for further analyses.

### 3.2 Population genetic diversity, structure, and differentiation

**Figure 2:**
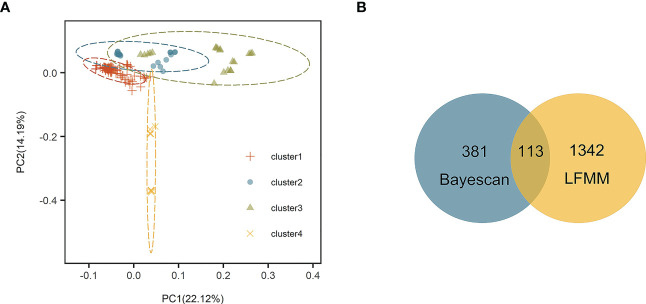
**(A)** Principal component analysis (PCA) based on 25,786 single nucleotide polymorphisms (SNPs). **(B)** Venn diagram showing overlap of SNPs identified by Bayescan and latent factor mixed modeling (LFMM).

*Stipa breviflora* showed low genetic diversity at the species level, with a value of 0.1284 (*He*). Population HN, located in the central part of the *S. breviflora* distribution region, showed the highest genetic diversity with the highest *Ho* (0.2180), *He* (0.1919), and *π* (0.1146). In contrast, the peripheral population, PL, exhibited the lowest level of genetic diversity, with an *Ho* of 0.0076 and *He* of 0.0068 (**Table 1**). Admixture analysis indicated that *K* = 4 is the most likely number of genetic clusters according to the lowest cross-validation error value (**Figure S1A**). These four identified genetic clusters were largely consistent with their geographic distributions (**Figure 1**). All eastern populations formed Cluster 1. Cluster 2 was composed of populations from the northeastern QTP and the northern Tianshan Mountains, except for population TK. Cluster 3 included populations in the northern Tianshan Mountains and the northern Kunlun Mountains. The remaining two populations (PL and LZ) formed cluster 4. Using PCA, principal components 1 and 2 explained 22.12% and 14.19% of the total genetic variance, respectively, separated clusters 1 and 4 from other clusters, while clusters 2 and 3 were mixed (**Figure 2A**). As **Table 2** indicates, the majority of variations (57.01%, *p *< 0.001) were explained among individuals within populations. Variation between groups accounted for only 17.99% of the total variance (*p *< 0.001), and a higher percentage of variance (25.00%, *p *< 0.001) was attributed to the difference among populations within groups. We detected a large genetic differentiation between populations, with *F_ST_* values ranging from 0.04 (BN vs. HG) to 0.36 (PL vs. HJ) (**Table S3**).

**Table 2:** The analysis of molecular variance (AMOVA) among 27 *S. breviflora* populations.

Source of variation	df	Sum of squares	Variance components	Percentage of variation (%)	Fixation index
Among groups	3	299.36	1.49	17.99	0.18*
Among populations within groups	23	585.00	2.07	25.00	0.30*
Within populations	243	1147.80	4.72	57.01	0.43*
Total	269	2032.16	8.29		

df, degrees of freedom; *p < 0.01.

The Mantel test detected significant patterns in both isolation by distance (IBD) (*r* = 0.633, *p* < 0.01) and isolation by environment (IBE) (*r* = 0.625, *p* < 0.01) in *S. breviflora* populations, which were also supported by a partial Mantel test (IBD: *r* = 0.411, *p* < 0.01; IBE: *r* = 0.392, *p* < 0.01) and MMRR (IBD: *β* = 0.127, *p* < 0.01; IBE: *β* = 0.063, *p* < 0.01) (**Table 3**). These analyses suggested significant effects of both IBD and IBE on the divergence of *S. breviflora* populations. IBD explains slightly more of the variation in genetic differentiation than IBE.

**Table 3:** Results of the Mantel test, partial Mantel test, and multiple matrix regression with randomization (MMRR).

	Mantel test		Partial Mantel test		MMRR	
	*r*	*p*	*r*	*p*	*β*	*p*
IBD	0.633	1.00E-04*	0.411	1.00E-04*	0.127	1.00E-04*
IBE	0.625	1.00E-04*	0.392	4.00E-04*	0.063	9.00E-04*

IBD, isolation by distance; IBE, isolation by environment; *p < 0.01.

### 3.3 Outlier analyses

Bayescan and LFMM analysis detected 494 and 1,455 outlier SNPs, respectively. There are 113 overlapping outlier SNPs were identified as outlier dataset (**Figure 2B**). Among them, three SNPs (loci 20733068, 17208702, and 17208770) were matched to two annotated contigs (receptor-like protein kinase 2 and common plant regulatory factor 1) in the *S. breviflora* transcriptome with an *E* value < 10^–5^ (**Table 4**). Locus 20733068 matched to receptor-like protein kinase 2 (*RPK2*; GO term: protein kinase activity; protein binding; ATP binding), which regulates plant growth (Mizuno et al., 2007). Loci 17208702 and 17208770 matched to common plant regulatory factor 1 (CPRF1; GO term: DNA-binding transcription factor activity; sequence-specific DNA binding), which relates to the light response of the plant (**Table 4**; **Table S4**).

**Table 4:** Annotation information of the three candidate loci selected.

Loci ID	Gene name	Description	Length (bp)	*E*-value
20733068	RPK2	Receptor-like protein kinase 2	146	1.47E-72
17208702;17208770	CPRF1	Common plant regulatory factor 1	146	1.47E-72

### 3.4 Genetic variation associated with environmental factors

**Figure 3:**
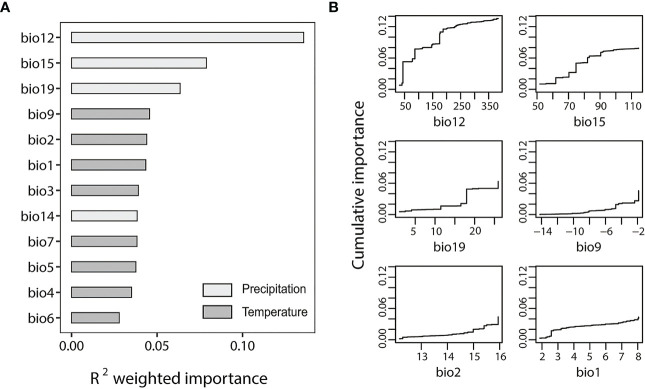
Gradient forest (GF) analysis of outlier-single nucleotide polymorphism (SNP) dataset. **(A)** *R*^2^-weighted importance of environmental variables. **(B)** Cumulative importance of allelic change along the first six environmental gradients. The bioclimatic factors included both in figure **(A, B)** are as follows: bio1, annual mean temperature; bio2, mean diurnal range; bio3 isothermality; bio4 temperature seasonality; bio5, max temperature of warmest month; bio6, minimum temperature of coldest month; bio7, temperature annual range; bio9, mean temperature of driest month; bio12, annual precipitation; bio14, precipitation of driest month; bio15, precipitation seasonality; and bio19, precipitation of coldest quarter.

We used the all-SNP dataset and outlier-SNP dataset to perform GF analyses to test the environmental effects on the population divergence and local adaptation of *S. breviflora*. The outlier-SNP dataset (**Figure 3A**) showed an overall higher *R*^2^ weighted importance value than the all-SNP dataset (**Figure S2A**). **Figures 3B**; **Figure S2B** indicate the cumulative importance of all allele frequency changes with the environmental gradient. Annual precipitation (bio12) showed a strong correlation with SNPs included in both of the two datasets. ANOVA analysis of annual precipitation (bio12) indicated a significant difference among four genetic clusters (*F* = 7.36; *p* < 0.05), demonstrating the considerable heterogeneity of precipitation in the study region. However, temperature factors appeared to be more highly associated with genetic differentiation for the all-SNP dataset, with bio2 (mean diurnal range), bio3 (isothermality), and bio1 (annual mean temperature) ranking second, third, and fourth in *R*^2^ weighted importance, respectively (**Figure S2A**). For the outlier-SNP dataset, annual precipitation (bio12), precipitation seasonality (bio15), and precipitation of the coldest quarter (bio19) are important (**Figure 3A**).

## 4 Discussion

### 4.1 Genetic diversity, structure, and differentiation

We detected a low level of genetic diversity in *S. breviflora* (*He* = 0.1284) using RAD sequencing data (**Table 1**). This is in sharp contrast to previous studies by Yan et al. (2020) (*He* = 0.34) and Ren et al. (2022) (*He* = 0.52), which were based on simple-sequence repeat (SSR) markers. A similar phenomenon occurs in the study of maize germplasm (*He* = 0.263 and *He* = 0.77 based on SNPs and SSRs, respectively) (Taramino and Tingey, 1996; Rafalski, 2002). This result may be due to the differences in molecular marker systems. SSR allelic diversity is generated by replication slippage, which contributes to the multiallelic characteristic of SSR markers (Gupta and Varshney, 2000; Varshney et al., 2005). SNP markers are mainly biallelic and consequently exhibit less information than SSR markers (Varshney et al., 2007). In addition, our results demonstrated a spatial configuration of decreasing genetic diversity from the center to the peripheral populations, as indicated by the trend of *Ho*, *He*, and π (**Table 1**). This center-to-edge pattern of genetic diversity has also been observed in many other species, such as *Emmenopterys henryi* (Xu et al., 2021), *Euptelea pleiosperma* (Wei et al., 2016), and *Taxus wallichiana* var. *mairei* (Liu et al., 2019).

Our admixture analysis identified four optimal clusters along the distribution range of *S. breviflora* (**Figure 1A**), and this was also supported by PCA results (**Figure 2A**). These clusters almost entirely corresponded to different geographic regions. Western populations were mainly distributed in the Mongolian Plateau and Loess Plateau, which are relatively flat with no large geographical barriers to gene flow. In contrast, eastern populations were geographically separated by the Tianshan Mountains and the QTP, which likely act not only as dispersal boundaries, limiting the gene flow of *S. breviflora*, but also create the complicated and heterogeneous habitats that promote local adaptation, shaping the spatial pattern of the genetic structure. In our recorded observations, we noticed that *S. breviflora* tends to have longer reproductive branches in regions with high precipitation levels. Moreover, we observed the purple spikelet of the *S. breviflora* in PL, which is distinct from the green, yellow–green, or pale-yellow (depending on the phenological stage) spikelet in other populations. We speculated that the distinctive purple spikelet is an adaptive trait that enables the species to survive in a harsh environment with strong solar radiation in the QTP. Both IBD and IBE played important roles in triggering genetic differentiation in *S. breviflora* (**Table 3**). However, only significant IBD was detected in the previous studies of Zhang et al. (2012) and Ren et al. (2022). This may be because a great number of SNPs were used in the present study, which provides more informative loci to explain the genetic differentiation of *S. breviflora*. In addition, PCA demonstrated mixed phenomena among populations TK, AK, and HJ (**Figure 1A**; **Figure S3**). We suggest that a future study should use a larger sample size for TK, AK, and HJ, to obtain clearer clustering results, as AMOVA results indicate that genetic variation within populations accounted for the majority (57.01%) of variations in *S. breviflora* (**Table 1**).

### 4.2 Impacts of environmental heterogeneity on population divergence and local adaptation

Annual precipitation (bio12) was detected as the most important climatic variable in the two datasets (**Figure 3**; **Figure S2**), suggesting that precipitation is an important driver of the genetic variation and local adaptation of *S. breviflora* populations. However, the increasing pattern of bio12’s cumulative importance was different between datasets. It showed a gradual increase for the all-SNP dataset (**Figure S2B**); however, a step-like increase at 50 mm, 100 mm, and 200 mm of precipitation was observed for the outlier-SNPs dataset (**Figure 3B**), suggesting that the outlier frequency of *S. breviflora* changed to adapt to precipitation changes in the regions with rainfalls of 50 mm, 100 mm, and 200 mm. The effects of temperature factors such as mean diurnal range (bio2), isothermality (bio3), and annual mean temperature (bio1) were also noticeable, as **Figure S2** shows. Our finding that temperature plays an important role in the genetic differentiation of *S. breviflora* populations is consistent with the results of a previous study by Zhang et al. (2012). *Stipa breviflora* is mainly distributed in temperate eastern Asia, where there are temporal and spatial variations in rainfall, confined mainly to summer (Dore, 2005). Therefore, it is not surprising to find that both precipitation and temperature contributed to the population differentiation of *S. breviflora* in the present study, as precipitation and temperature are often linearly related to the distribution areas of plants. Although most studies use GF analysis to explore the relationship between environmental and genetic differentiation, we combined the methods of *F_ST_* outlier tests and EAA, and selected outliers for GF analysis, providing a new insight into the mechanism for *S. breviflora* local adaptation, i.e., that precipitation plays a key role in the process of local adaptation.

### 4.3 Genomic signatures associated with local adaptation

We obtained the gene *RPK2* and transcription factor CPRF1 by blasting outlier loci to the transcriptome data of *S. breviflora*. *RPK2* is a regulator of plant meristem maintenance (Kinoshita et al., 2010) and plays a part in anther and embryo development (Mizuno et al., 2007). This gene is involved in the signaling pathway CLAVATA 3 (CLV3), which controls stem renewal and differentiation (Shimizu et al., 2015; Shinohara and Matsubayashi, 2015). Considering that *S. breviflora* lives in a highly heterogeneous and complicated environment, we speculate that *RPK2* may contribute to balance cell proliferation and differentiation to help it survive in harsh environments. CPRF1 is a transcription factor involved in the regulation of chalcone synthase (*CHS*) gene expression, which is responsible for light responsiveness (Feldbrügge et al., 1994; Jiao et al., 2007). UV light induces synthesis of CPRF1, which activates the expression of the light-responsive *CHS* gene, a key gene in the biosynthesis of flavonoids that protect plants against the damaging effects of UV irradiation (Strid et al., 1994; Sprenger-Haussels and Weisshaar, 2000; Zhang et al., 2018). We suggest that CPRF1 is of importance in facilitating the adaptation of *S. breviflora* to the intense UV radiation found in the QTP region.

There were some limits to our study. Firstly, given that RADseq covers only the partial genome of *S. breviflora*, we could obtain only a portion of the loci involved in adaptation. Secondly, the lack of whole-genome data for *S. breviflora* also brings bias and inaccuracy to the annotation of outliers. Thirdly, genetic variations that relate to local adaptation are most likely polygenic and controlled by numerous small-effect genes (Savolainen et al., 2013), and it is still challenging for most methods to detect loci with small or moderate effects (Wellenreuther and Hansson, 2016). Finally, pleiotropy is a common phenomenon in which a mutation in one gene can affect more than one phenotypic character. In the present study, it is possible that a single adaptive locus may be associated with multiple phenotypes. However, without sufficient phenotypic information, it is difficult to link specific traits to their underlying genetic mutations. To establish these connections, genome-wide association studies (GWAS) should be considered for future studies.

## Data availability statement

The data presented in the study are deposited in the GSA (Genome Sequence Archive) of NGDC (National Genomics Data Center) repository, accession number CRA007694.

## Author contributions

JN contributed to the conception and design of the study. JN, DY, JL, YF, and ZL contributed to the investigation of the study. ZD, DY, and JL performed the statistical analysis. DY and JL performed the visualization of the study. DY wrote the original draft of the manuscript. JN revised and edited the manuscript. All authors contributed to the article and approved the submitted version.
