# Supplementary material for: Genomic insights into genetic diversity and local adaptation of a dominant desert steppe feather grass, Stipa breviflora Griseb
Source: Front Plant Sci. 2023 May 17;14:1170075. doi: 10.3389/fpls.2023.1170075 (PMC10230062; doi:10.3389/fpls.2023.1170075)
Supplement: Supplementary file 1 [file DataSheet_1.docx]

***Supplementary Material***

**Genomic insights into genetic diversity and local adaptation of a dominant desert steppe feather grass, *Stipa breviflora* Griesb.**

**Dongqing Yan*, Jiamei Liu, Yanyan Fan, Zhi Lian**

***Correspondence:** Zhenhua Dang: zhdang_1982@aliyun.com

Jianming Niu: [jmniu2005@163.com](mailto:jmniu2005@163.com)

**Table S1 Nineteen bioclimatic data corresponding those from Worldclim of each population**

| Population ID | **bio1**  **(℃)** | **bio2**  **(℃)** | **bio3**  **(%)** | **bio4**  **（SD）** | **bio5**  **(℃)** | **bio6**  **(℃)** | **bio7**  **(℃)** | bio8  (℃) | **bio9**  **(℃)** | bio10  (℃) |
| --- | --- | --- | --- | --- | --- | --- | --- | --- | --- | --- |
| WN | **7.6** | **12.9** | **28.1** | **1279.9** | **28.9** | **-16.8** | **45.7** | 22.5 | **-6.6** | 22.5 |
| BL | **3.3** | **12.9** | **26.5** | **1350.5** | **26.6** | **-22.0** | **48.6** | 19.1 | **-14.2** | 19.1 |
| BT | **3.6** | **12.3** | **25.4** | **1367.1** | **26.9** | **-21.5** | **48.4** | 19.6 | **-14.1** | 19.6 |
| BN | **4.1** | **13.0** | **27.0** | **1307.0** | **27.0** | **-21.0** | **48.0** | 19.6 | **-12.7** | 19.6 |
| HG | **4.0** | **13.0** | **27.1** | **1295.4** | **26.4** | **-21.6** | **48.0** | 19.1 | **-12.8** | 19.1 |
| ZG | **6.6** | **13.9** | **29.2** | **1233.2** | **28.8** | **-18.8** | **47.6** | 19.0 | **-9.6** | 20.9 |
| BY | **5.2** | **13.6** | **27.7** | **1320.5** | **28.7** | **-20.4** | **49.1** | 18.8 | **-11.9** | 20.8 |
| CJ | **5.0** | **12.6** | **26.5** | **1303.9** | **28.0** | **-19.7** | **47.7** | 20.4 | **-11.8** | 20.4 |
| HQ | **6.9** | **14.0** | **29.7** | **1205.0** | **29.2** | **-17.9** | **47.1** | 19.3 | **-8.6** | 21.1 |
| ET | **7.0** | **14.2** | **30.5** | **1182.0** | **29.1** | **-17.4** | **46.5** | 19.4 | **-8.1** | 21.0 |
| HX | **8.0** | **13.2** | **31.6** | **1030.3** | **27.6** | **-14.1** | **41.7** | 18.7 | **-5.3** | 20.3 |
| WZ | **8.1** | **13.4** | **31.9** | **1046.3** | **28.2** | **-14.0** | **42.2** | 18.9 | **-5.4** | 20.5 |
| SH | **7.5** | **14.2** | **30.7** | **1187.6** | **29.7** | **-16.5** | **46.2** | 21.6 | **-7.7** | 21.6 |
| GL | **8.0** | **13.0** | **31.7** | **1002.0** | **27.3** | **-13.6** | **40.9** | 18.3 | **-5.1** | 19.7 |
| ML | **2.2** | **14.8** | **34.8** | **998.0** | **21.6** | **-20.9** | **42.5** | 13.8 | **-9.4** | 13.8 |
| AK | **2.3** | **14.5** | **35.1** | **1013.7** | **22.4** | **-19.0** | **41.4** | 14.6 | **-4.4** | 14.6 |
| BK | **1.6** | **12.3** | **27.4** | **1237.7** | **23.3** | **-21.5** | **44.8** | 16.1 | **-14.2** | 16.1 |
| HJ | **2.3** | **13.3** | **33.4** | **982.6** | **21.1** | **-18.7** | **39.8** | 13.6 | **-8.8** | 13.6 |
| TK | **7.1** | **12.9** | **28.4** | **1188.0** | **28.0** | **-17.3** | **45.3** | 18.8 | **-8.9** | 20.2 |
| TL | **4.3** | **12.1** | **26.9** | **1242.1** | **27.1** | **-17.9** | **45.0** | 17.0 | **-11.5** | 19.0 |
| KS | **3.1** | **13.2** | **31.9** | **1022.4** | **23.5** | **-18.0** | **41.5** | 12.9 | **-8.4** | 14.7 |
| YC | **5.8** | **13.9** | **34.2** | **989.8** | **25.9** | **-14.7** | **40.6** | 17.3 | **-0.3** | 17.3 |
| MF | **6.1** | **16.2** | **37.2** | **1049.6** | **26.9** | **-16.7** | **43.6** | 16.6 | **-4.4** | 18.1 |
| HN | **2.8** | **15.2** | **37.7** | **902.3** | **20.6** | **-19.6** | **40.2** | 13.3 | **-8.1** | 13.3 |
| DL | **3.5** | **14.7** | **35.8** | **969.4** | **22.6** | **-18.6** | **41.2** | 14.8 | **-7.9** | 14.8 |
| LZ | **4.6** | **15.6** | **47.5** | **635.3** | **18.8** | **-14.1** | **32.9** | 12.1 | **-3.4** | 12.1 |
| PL | **4.2** | **13.5** | **43.1** | **695.2** | **19.1** | **-12.3** | **31.4** | 11.6 | **8.4** | 12.2 |

Continued

| Population ID | bio11  (℃) | **bio12**  **(mm)** | bio13  (mm) | **bio14**  **(mm)** | **bio15**  **(%)** | bio16  (mm) | bio17  (mm) | bio18  (mm) | **bio19**  **(mm)** |
| --- | --- | --- | --- | --- | --- | --- | --- | --- | --- |
| WN | -8.9 | **375** | 113 | **1** | **113.3** | 259 | 6 | 259 | **6** |
| BL | -14.2 | **325** | 89 | **1** | **109.2** | 219 | 6 | 219 | **6** |
| BT | -14.1 | **289** | 80 | **1** | **110.6** | 197 | 5 | 197 | **5** |
| BN | -12.7 | **205** | 63 | **1** | **113.7** | 140 | 5 | 140 | **5** |
| HG | -12.8 | **198** | 63 | **1** | **115.2** | 135 | 5 | 135 | **5** |
| ZG | -9.6 | **384** | 106 | **3** | **110.1** | 254 | 10 | 248 | **10** |
| BY | -11.9 | **237** | 65 | **2** | **106.2** | 154 | 9 | 153 | **9** |
| CJ | -11.8 | **220** | 54 | **3** | **91.3** | 131 | 10 | 131 | **10** |
| HQ | -8.6 | **266** | 77 | **2** | **109.4** | 178 | 7 | 169 | **7** |
| ET | -8.1 | **246** | 70 | **2** | **99.4** | 153 | 9 | 147 | **9** |
| HX | -5.3 | **341** | 82 | **1** | **92.3** | 204 | 6 | 185 | **6** |
| WZ | -5.4 | **279** | 71 | **1** | **94.9** | 169 | 5 | 157 | **5** |
| SH | -7.7 | **122** | 32 | **0** | **97.6** | 77 | 2 | 77 | **2** |
| GL | -5.1 | **326** | 75 | **1** | **92.2** | 188 | 5 | 182 | **5** |
| ML | -10.7 | **352** | 80 | **2** | **101.3** | 217 | 7 | 217 | **7** |
| AK | -10.4 | **53** | 15 | **1** | **89.6** | 36 | 3 | 36 | **3** |
| BK | -14.2 | **177** | 37 | **2** | **74.8** | 95 | 8 | 95 | **8** |
| HJ | -10.4 | **230** | 53 | **1** | **104.2** | 145 | 3 | 145 | **3** |
| TK | -8.9 | **228** | 36 | **9** | **45.5** | 96 | 29 | 91 | **29** |
| TL | -11.5 | **269** | 45 | **7** | **58.1** | 125 | 23 | 115 | **23** |
| KS | -10.3 | **173** | 30 | **3** | **65.8** | 83 | 12 | 78 | **13** |
| YC | -7.1 | **30** | 9 | **0** | **74.6** | 17 | 1 | 17 | **3** |
| MF | -7.7 | **35** | 11 | **0** | **100.1** | 26 | 0 | 25 | **0** |
| HN | -8.8 | **383** | 91 | **1** | **100.7** | 236 | 6 | 236 | **6** |
| DL | -9.0 | **178** | 40 | **1** | **93.5** | 112 | 8 | 112 | **10** |
| LZ | -3.4 | **286** | 76 | **2** | **107.5** | 190 | 7 | 190 | **7** |
| PL | -4.7 | **840** | 198 | **15** | **86.2** | 470 | 77 | 409 | **177** |

Note: The unit of each bioclimatic variables is provided in the parenthesis. Bio1, annual mean temperature; bio2, mean diurnal range; bio3, isothermality; bio4, temperature seasonality; bio5, max temperature of warmest month; bio6, min temperature of coldest month; bio7, temperature annual range; bio8, mean temperature of wettest quarter; bio9, mean temperature of driest quarter; bio10, mean temperature of warmest quarter; bio11, mean temperature of coldest quarter; bio12, annual precipitation; bio13, precipitation of wettest month; bio14, precipitation of driest month; bio15, precipitation seasonality; bio16, precipitation of wettest quarter; bio17, precipitation of driest quarter; bio18, precipitation of warmest quarter; bio19, precipitation of coldest quarter; SD, standard deviation. 12 bioclimatic variables used in GF analysis are in bold.

**Table S2 Summary of quality of RAD-seq for each individual of *S. breviflora***

| Population | Sample ID | Clean reads (M) | Base number (G) | GC  (%) | Q20  (%) | Q30  (%) |
| --- | --- | --- | --- | --- | --- | --- |
| WN | J1_1 | 19.44 | 2.79 | 43.80 | 97.61 | 93.87 |
|  | J1_2 | 18.55 | 2.73 | 42.67 | 98.39 | 95.58 |
|  | J1_3 | 22.80 | 3.35 | 42.50 | 98.43 | 95.66 |
|  | J1_4 | 22.27 | 3.30 | 42.97 | 98.40 | 95.60 |
|  | J1_5 | 23.34 | 3.44 | 42.62 | 98.44 | 95.68 |
| BL | J2_1 | 21.40 | 3.16 | 42.69 | 98.43 | 95.65 |
|  | J2_2 | 22.07 | 3.23 | 42.79 | 98.40 | 95.58 |
|  | J2_3 | 21.86 | 3.19 | 42.70 | 98.39 | 95.57 |
|  | J2_4 | 21.25 | 3.10 | 42.74 | 98.41 | 95.60 |
|  | J2_5 | 19.87 | 2.92 | 42.75 | 98.33 | 95.42 |
| BT | J3_1 | 23.09 | 3.39 | 42.55 | 98.36 | 95.49 |
|  | J3_2 | 25.16 | 3.72 | 42.63 | 98.36 | 95.51 |
|  | J3_3 | 23.46 | 3.46 | 42.67 | 98.37 | 95.53 |
|  | J3_4 | 24.01 | 3.54 | 42.46 | 98.38 | 95.55 |
|  | J3_5 | 21.95 | 3.22 | 42.52 | 98.34 | 95.46 |
| BN | J4_1 | 24.57 | 3.59 | 42.77 | 98.33 | 95.43 |
|  | J4_2 | 22.50 | 3.29 | 42.68 | 98.35 | 95.48 |
|  | J4_3 | 18.78 | 2.76 | 42.72 | 98.19 | 95.08 |
|  | J4_4 | 19.59 | 2.88 | 42.88 | 98.21 | 95.12 |
|  | J4_5 | 17.69 | 2.62 | 42.83 | 98.21 | 95.13 |
| HG | J5_1 | 19.62 | 2.89 | 42.71 | 98.22 | 95.16 |
|  | J5_2 | 21.11 | 3.11 | 42.65 | 98.25 | 95.23 |
|  | J5_3 | 19.08 | 2.80 | 42.69 | 98.20 | 95.10 |
|  | J5_4 | 19.12 | 2.79 | 42.61 | 98.19 | 95.08 |
|  | J5_5 | 18.56 | 2.71 | 42.61 | 98.21 | 95.13 |
| ZG | J6_1 | 25.37 | 3.63 | 43.44 | 97.68 | 94.03 |
|  | J6_2 | 15.15 | 2.23 | 42.80 | 98.27 | 95.30 |
|  | J6_3 | 20.01 | 2.94 | 42.87 | 98.32 | 95.40 |
|  | J6_4 | 18.63 | 2.76 | 42.67 | 98.32 | 95.40 |
|  | J6_5 | 18.56 | 2.74 | 42.74 | 98.33 | 95.43 |
| BY | J7_1 | 18.31 | 2.70 | 42.78 | 98.32 | 95.40 |
|  | J7_2 | 19.79 | 2.90 | 42.85 | 98.29 | 95.32 |
|  | J7_3 | 20.78 | 3.03 | 42.81 | 98.28 | 95.31 |
|  | J7_4 | 18.54 | 2.71 | 42.82 | 98.30 | 95.36 |
|  | J7_5 | 15.25 | 2.24 | 42.79 | 98.35 | 95.48 |
| CJ | J8_1 | 17.17 | 2.52 | 42.69 | 98.37 | 95.52 |
|  | J8_2 | 21.88 | 3.24 | 42.68 | 98.38 | 95.54 |
|  | J8_3 | 22.33 | 3.29 | 42.69 | 98.40 | 95.59 |
|  | J8_4 | 24.81 | 3.66 | 42.57 | 98.40 | 95.61 |
|  | J8_5 | 18.60 | 2.72 | 42.65 | 98.36 | 95.50 |
| HQ | J9_1 | 23.44 | 3.42 | 42.69 | 98.36 | 95.51 |
|  | J9_2 | 21.18 | 3.09 | 42.69 | 98.38 | 95.54 |
|  | J9_3 | 19.65 | 2.89 | 43.95 | 98.30 | 95.37 |
|  | J9_4 | 23.05 | 3.39 | 44.03 | 98.33 | 95.43 |
|  | J9_5 | 22.25 | 3.29 | 44.15 | 98.33 | 95.42 |
| ET | J10_1 | 23.73 | 3.50 | 43.98 | 98.35 | 95.47 |
|  | J10_2 | 22.42 | 3.31 | 44.12 | 98.34 | 95.46 |
|  | J10_3 | 21.46 | 3.14 | 44.06 | 98.32 | 95.39 |
|  | J10_4 | 22.90 | 3.34 | 43.98 | 98.31 | 95.38 |
|  | J10_5 | 21.14 | 3.09 | 44.09 | 98.33 | 95.42 |
| HX | J11_1 | 12.84 | 1.89 | 44.05 | 98.34 | 95.44 |
|  | J11_2 | 14.89 | 2.19 | 44.05 | 98.36 | 95.50 |
|  | J11_3 | 14.14 | 2.09 | 44.21 | 98.36 | 95.49 |
|  | J11_4 | 16.28 | 2.40 | 44.10 | 98.38 | 95.55 |
|  | J11_5 | 15.05 | 2.22 | 43.99 | 98.38 | 95.55 |
| WZ | J12_1 | 15.88 | 2.33 | 43.89 | 98.37 | 95.52 |
|  | J12_2 | 15.54 | 2.27 | 43.94 | 98.35 | 95.47 |
|  | J12_3 | 15.76 | 2.30 | 43.90 | 98.38 | 95.54 |
|  | J12_4 | 15.69 | 2.31 | 43.96 | 99.01 | 97.23 |
|  | J12_5 | 19.91 | 2.93 | 43.91 | 99.04 | 97.30 |
| SH | J13_1 | 15.35 | 2.20 | 43.47 | 97.65 | 93.99 |
|  | J13_2 | 16.61 | 2.46 | 43.97 | 99.03 | 97.29 |
|  | J13_3 | 18.89 | 2.79 | 43.82 | 99.05 | 97.32 |
|  | J13_4 | 17.00 | 2.51 | 43.81 | 99.05 | 97.32 |
|  | J13_5 | 20.45 | 3.00 | 43.89 | 99.03 | 97.28 |
| GL | J14_1 | 22.04 | 3.16 | 44.04 | 97.67 | 94.01 |
|  | J14_4 | 17.58 | 2.57 | 43.87 | 99.03 | 97.29 |
|  | J14_5 | 15.82 | 2.31 | 44.00 | 99.05 | 97.33 |
|  | J14_9 | 17.71 | 2.61 | 42.98 | 99.03 | 97.29 |
|  | J14_10 | 15.45 | 2.28 | 43.12 | 99.02 | 97.27 |
| ML | J15_1 | 24.38 | 3.52 | 43.61 | 97.72 | 94.13 |
|  | J15_2 | 16.81 | 2.47 | 44.11 | 99.04 | 97.29 |
|  | J15_3 | 21.51 | 3.16 | 43.97 | 99.06 | 97.36 |
|  | J15_4 | 21.96 | 3.25 | 44.00 | 99.06 | 97.34 |
|  | J15_5 | 22.36 | 3.30 | 43.83 | 99.07 | 97.37 |
| AK | J16_1 | 16.35 | 3.29 | 43.23 | 96.91 | 92.46 |
|  | J16_2 | 19.44 | 2.87 | 43.83 | 99.07 | 97.37 |
|  | J16_3 | 19.90 | 2.92 | 43.67 | 99.06 | 97.34 |
|  | J16_4 | 20.52 | 3.00 | 43.80 | 99.05 | 97.33 |
|  | J16_5 | 18.36 | 2.68 | 43.77 | 99.06 | 97.35 |
| BK | J17_1 | 19.15 | 2.74 | 43.39 | 97.72 | 94.13 |
|  | J17_3 | 20.73 | 3.05 | 43.66 | 99.00 | 97.21 |
|  | J17_4 | 25.51 | 3.75 | 43.64 | 99.03 | 97.28 |
|  | J17_5 | 22.59 | 3.34 | 43.70 | 99.03 | 97.27 |
|  | J17_6 | 24.85 | 3.66 | 43.69 | 99.05 | 97.31 |
| HJ | J18_1 | 16.87 | 2.41 | 43.39 | 97.62 | 93.89 |
|  | J18_2 | 23.15 | 3.41 | 43.43 | 99.04 | 97.31 |
|  | J18_3 | 23.65 | 3.46 | 43.77 | 99.03 | 97.26 |
|  | J18_4 | 21.77 | 3.18 | 43.57 | 99.02 | 97.25 |
|  | J18_5 | 18.73 | 2.73 | 43.03 | 99.06 | 97.34 |
| TK | J19_1 | 14.51 | 2.07 | 43.40 | 97.68 | 94.05 |
|  | J19_2 | 16.87 | 2.48 | 43.90 | 98.93 | 97.01 |
|  | J19_3 | 20.19 | 2.97 | 43.83 | 98.96 | 97.08 |
|  | J19_4 | 21.33 | 3.16 | 43.92 | 98.95 | 97.07 |
|  | J19_5 | 22.64 | 3.34 | 43.92 | 98.97 | 97.10 |
| TL | J20_1 | 23.38 | 3.33 | 43.64 | 97.76 | 94.19 |
|  | J20_2 | 20.92 | 3.09 | 43.55 | 98.98 | 97.13 |
|  | J20_3 | 21.88 | 3.21 | 43.84 | 98.95 | 97.06 |
|  | J20_4 | 20.19 | 2.95 | 43.79 | 98.94 | 97.05 |
|  | J20_5 | 18.05 | 2.64 | 43.78 | 98.96 | 97.09 |
| KS | J21_1 | 14.25 | 2.05 | 43.59 | 96.90 | 92.41 |
|  | J21_2 | 16.61 | 2.44 | 42.95 | 99.01 | 97.25 |
|  | J21_3 | 18.06 | 2.66 | 42.84 | 99.03 | 97.30 |
|  | J21_4 | 15.40 | 2.26 | 42.83 | 99.04 | 97.31 |
|  | J21_5 | 17.47 | 2.59 | 42.96 | 99.03 | 97.28 |
| YC | J22_1 | 12.51 | 1.80 | 43.64 | 96.86 | 92.32 |
|  | J22_2 | 18.30 | 2.70 | 42.87 | 99.05 | 97.33 |
|  | J22_3 | 16.51 | 2.44 | 42.82 | 99.04 | 97.32 |
|  | J22_4 | 17.96 | 2.63 | 43.07 | 99.03 | 97.28 |
|  | J22_5 | 15.78 | 2.30 | 42.85 | 99.02 | 97.26 |
| MF | J23_1 | 18.86 | 2.73 | 43.55 | 96.92 | 92.46 |
|  | J23_2 | 15.74 | 2.32 | 42.78 | 99.03 | 97.28 |
|  | J23_3 | 14.94 | 2.20 | 42.53 | 99.05 | 97.33 |
|  | J23_4 | 14.32 | 2.13 | 42.75 | 99.03 | 97.29 |
|  | J23_5 | 14.39 | 2.14 | 42.80 | 99.05 | 97.34 |
| HN | J24_1 | 18.14 | 2.61 | 44.12 | 96.88 | 92.36 |
|  | J24_6 | 13.53 | 2.01 | 43.88 | 99.02 | 97.26 |
|  | J24_7 | 12.07 | 1.79 | 44.24 | 99.01 | 97.23 |
|  | J24_8 | 12.36 | 1.83 | 44.22 | 99.02 | 97.25 |
|  | J24_9 | 11.38 | 1.69 | 44.14 | 99.04 | 97.31 |
| DL | J25_1 | 16.35 | 2.36 | 43.23 | 96.91 | 92.46 |
|  | J25_2 | 13.88 | 2.06 | 42.67 | 99.05 | 97.34 |
|  | J25_4 | 14.50 | 2.17 | 42.73 | 99.04 | 97.32 |
|  | J25_5 | 18.20 | 2.71 | 42.60 | 99.06 | 97.37 |
|  | J25_6 | 15.32 | 2.28 | 42.11 | 99.07 | 97.39 |
| LZ | J26_1 | 15.05 | 2.15 | 43.48 | 96.91 | 92.41 |
|  | J26_2 | 15.86 | 2.35 | 42.81 | 99.04 | 97.32 |
|  | J26_3 | 14.95 | 2.21 | 40.59 | 99.04 | 97.36 |
|  | J26_4 | 15.20 | 2.24 | 42.46 | 99.05 | 97.33 |
|  | J26_5 | 16.41 | 2.42 | 42.61 | 99.06 | 97.36 |
| PL | J27_1 | 17.00 | 2.42 | 43.61 | 96.86 | 92.31 |
|  | J27_2 | 15.92 | 2.37 | 42.86 | 98.98 | 97.17 |
|  | J27_3 | 18.64 | 2.77 | 42.93 | 99.00 | 97.21 |
|  | J27_4 | 15.04 | 2.23 | 42.98 | 99.00 | 97.21 |
|  | J27_5 | 16.20 | 2.42 | 43.11 | 99.00 | 97.20 |
| mean |  | 18.86 | 2.77 | 43.27 | 98.52 | 96.00 |

**Table S3 Pairwise genetic differentiation (*F_ST_*) of 27 *S. breviflora* populations**

| Population | WN | BL | BT | BN | HG | ZG | BY | CJ | HJ | ET | HX | WZ | SH |
| --- | --- | --- | --- | --- | --- | --- | --- | --- | --- | --- | --- | --- | --- |
| WN |  |  |  |  |  |  |  |  |  |  |  |  |  |
| BL | 0.10 |  |  |  |  |  |  |  |  |  |  |  |  |
| BT | 0.14 | 0.08 |  |  |  |  |  |  |  |  |  |  |  |
| BN | 0.12 | 0.07 | 0.08 |  |  |  |  |  |  |  |  |  |  |
| HG | 0.11 | 0.06 | 0.08 | 0.04 |  |  |  |  |  |  |  |  |  |
| ZG | 0.14 | 0.09 | 0.11 | 0.07 | 0.07 |  |  |  |  |  |  |  |  |
| BY | 0.14 | 0.09 | 0.11 | 0.06 | 0.06 | 0.06 |  |  |  |  |  |  |  |
| CJ | 0.14 | 0.10 | 0.12 | 0.07 | 0.07 | 0.08 | 0.06 |  |  |  |  |  |  |
| HJ | 0.17 | 0.12 | 0.14 | 0.09 | 0.10 | 0.09 | 0.08 | 0.09 |  |  |  |  |  |
| ET | 0.15 | 0.10 | 0.12 | 0.07 | 0.08 | 0.07 | 0.06 | 0.08 | 0.07 |  |  |  |  |
| HX | 0.14 | 0.09 | 0.12 | 0.07 | 0.07 | 0.07 | 0.07 | 0.08 | 0.09 | 0.07 |  |  |  |
| WZ | 0.17 | 0.12 | 0.14 | 0.10 | 0.10 | 0.09 | 0.09 | 0.11 | 0.11 | 0.09 | 0.09 |  |  |
| SH | 0.20 | 0.15 | 0.18 | 0.13 | 0.13 | 0.13 | 0.12 | 0.11 | 0.13 | 0.11 | 0.12 | 0.14 |  |
| GL | 0.15 | 0.09 | 0.11 | 0.06 | 0.07 | 0.07 | 0.07 | 0.07 | 0.09 | 0.08 | 0.06 | 0.10 | 0.11 |
| ML | 0.15 | 0.11 | 0.13 | 0.09 | 0.09 | 0.09 | 0.09 | 0.09 | 0.11 | 0.10 | 0.08 | 0.12 | 0.12 |
| AK | 0.22 | 0.16 | 0.19 | 0.14 | 0.14 | 0.15 | 0.14 | 0.14 | 0.17 | 0.15 | 0.14 | 0.18 | 0.15 |
| BK | 0.24 | 0.19 | 0.21 | 0.16 | 0.17 | 0.18 | 0.17 | 0.17 | 0.20 | 0.18 | 0.17 | 0.21 | 0.20 |
| HJ | 0.30 | 0.25 | 0.28 | 0.23 | 0.23 | 0.23 | 0.23 | 0.22 | 0.25 | 0.24 | 0.22 | 0.26 | 0.25 |
| TK | 0.26 | 0.20 | 0.22 | 0.18 | 0.18 | 0.19 | 0.17 | 0.17 | 0.20 | 0.18 | 0.17 | 0.21 | 0.17 |
| TL | 0.26 | 0.21 | 0.23 | 0.18 | 0.19 | 0.19 | 0.18 | 0.18 | 0.19 | 0.18 | 0.18 | 0.21 | 0.16 |
| KS | 0.31 | 0.25 | 0.28 | 0.24 | 0.23 | 0.24 | 0.23 | 0.22 | 0.25 | 0.24 | 0.23 | 0.27 | 0.23 |
| YC | 0.30 | 0.25 | 0.27 | 0.23 | 0.23 | 0.24 | 0.23 | 0.22 | 0.25 | 0.23 | 0.21 | 0.25 | 0.22 |
| MF | 0.34 | 0.29 | 0.31 | 0.27 | 0.27 | 0.27 | 0.26 | 0.25 | 0.28 | 0.26 | 0.25 | 0.29 | 0.25 |
| HN | 0.18 | 0.12 | 0.15 | 0.11 | 0.11 | 0.12 | 0.13 | 0.13 | 0.15 | 0.13 | 0.12 | 0.14 | 0.16 |
| DL | 0.20 | 0.15 | 0.17 | 0.13 | 0.13 | 0.13 | 0.12 | 0.12 | 0.15 | 0.13 | 0.12 | 0.16 | 0.15 |
| LZ | 0.29 | 0.25 | 0.26 | 0.23 | 0.23 | 0.23 | 0.23 | 0.23 | 0.25 | 0.23 | 0.21 | 0.25 | 0.25 |
| PL | 0.31 | 0.26 | 0.29 | 0.24 | 0.24 | 0.25 | 0.24 | 0.24 | 0.25 | 0.25 | 0.23 | 0.27 | 0.27 |

Continued

| Population | GL | ML | AK | BK | HJ | TK | TL | KS | YC | MF | HN | DL | LZ | PL |
| --- | --- | --- | --- | --- | --- | --- | --- | --- | --- | --- | --- | --- | --- | --- |
| WN |  |  |  |  |  |  |  |  |  |  |  |  |  |  |
| BL |  |  |  |  |  |  |  |  |  |  |  |  |  |  |
| BT |  |  |  |  |  |  |  |  |  |  |  |  |  |  |
| BN |  |  |  |  |  |  |  |  |  |  |  |  |  |  |
| HG |  |  |  |  |  |  |  |  |  |  |  |  |  |  |
| ZG |  |  |  |  |  |  |  |  |  |  |  |  |  |  |
| BY |  |  |  |  |  |  |  |  |  |  |  |  |  |  |
| CJ |  |  |  |  |  |  |  |  |  |  |  |  |  |  |
| HJ |  |  |  |  |  |  |  |  |  |  |  |  |  |  |
| ET |  |  |  |  |  |  |  |  |  |  |  |  |  |  |
| HX |  |  |  |  |  |  |  |  |  |  |  |  |  |  |
| WZ |  |  |  |  |  |  |  |  |  |  |  |  |  |  |
| SH |  |  |  |  |  |  |  |  |  |  |  |  |  |  |
| GL |  |  |  |  |  |  |  |  |  |  |  |  |  |  |
| ML | 0.07 |  |  |  |  |  |  |  |  |  |  |  |  |  |
| AK | 0.12 | 0.12 |  |  |  |  |  |  |  |  |  |  |  |  |
| BK | 0.17 | 0.17 | 0.18 |  |  |  |  |  |  |  |  |  |  |  |
| HJ | 0.21 | 0.21 | 0.16 | 0.29 |  |  |  |  |  |  |  |  |  |  |
| TK | 0.16 | 0.17 | 0.19 | 0.26 | 0.27 |  |  |  |  |  |  |  |  |  |
| TL | 0.18 | 0.19 | 0.22 | 0.20 | 0.31 | 0.25 |  |  |  |  |  |  |  |  |
| KS | 0.21 | 0.21 | 0.20 | 0.29 | 0.30 | 0.21 | 0.31 |  |  |  |  |  |  |  |
| YC | 0.20 | 0.20 | 0.17 | 0.30 | 0.26 | 0.26 | 0.30 | 0.16 |  |  |  |  |  |  |
| MF | 0.24 | 0.23 | 0.19 | 0.32 | 0.28 | 0.29 | 0.32 | 0.16 | 0.09 |  |  |  |  |  |
| HN | 0.10 | 0.10 | 0.13 | 0.19 | 0.22 | 0.21 | 0.22 | 0.22 | 0.19 | 0.23 |  |  |  |  |
| DL | 0.11 | 0.11 | 0.12 | 0.20 | 0.21 | 0.19 | 0.22 | 0.20 | 0.19 | 0.21 | 0.12 |  |  |  |
| LZ | 0.21 | 0.20 | 0.22 | 0.31 | 0.31 | 0.31 | 0.32 | 0.30 | 0.29 | 0.31 | 0.17 | 0.21 |  |  |
| PL | 0.22 | 0.22 | 0.27 | 0.34 | 0.36 | 0.32 | 0.34 | 0.34 | 0.29 | 0.35 | 0.23 | 0.25 | 0.31 |  |

**Table S4 detailed annotation information of the two genes corresponding those in Table 4**

| Loci ID | Gene names | Description | GO IDs | GO names |
| --- | --- | --- | --- | --- |
| 20733068 | RPK2 | receptor-like protein kinase 2 | GO:0004672 | protein kinase activity |
|  |  |  | GO:0005515 | protein binding |
|  |  |  | GO:0005524 | ATP binding |
| 17208702 17208770 | CPRF1 | common plant regulatory factor 1 | GO:0003700 | DNA-binding transcription factor activity |
|  |  |  | GO:0043565 | sequence-specific DNA binding |

(A)


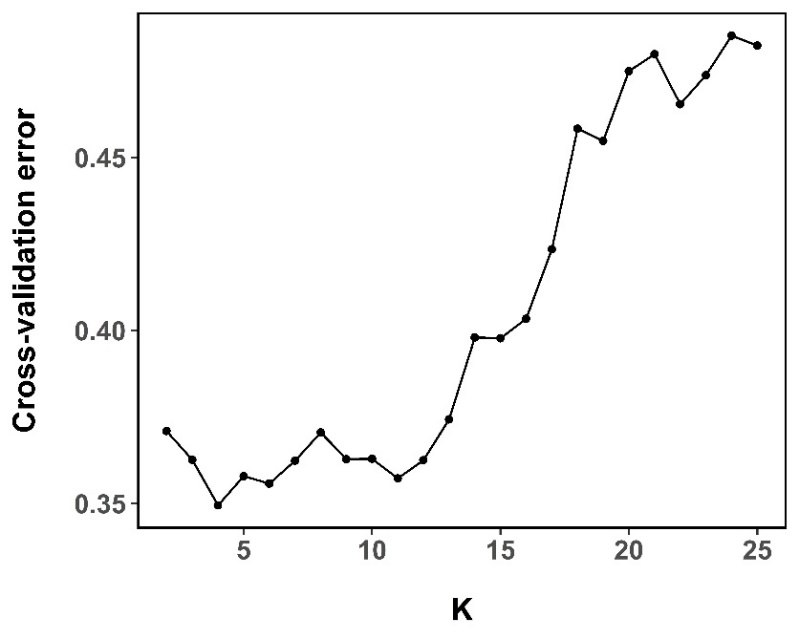


(B)


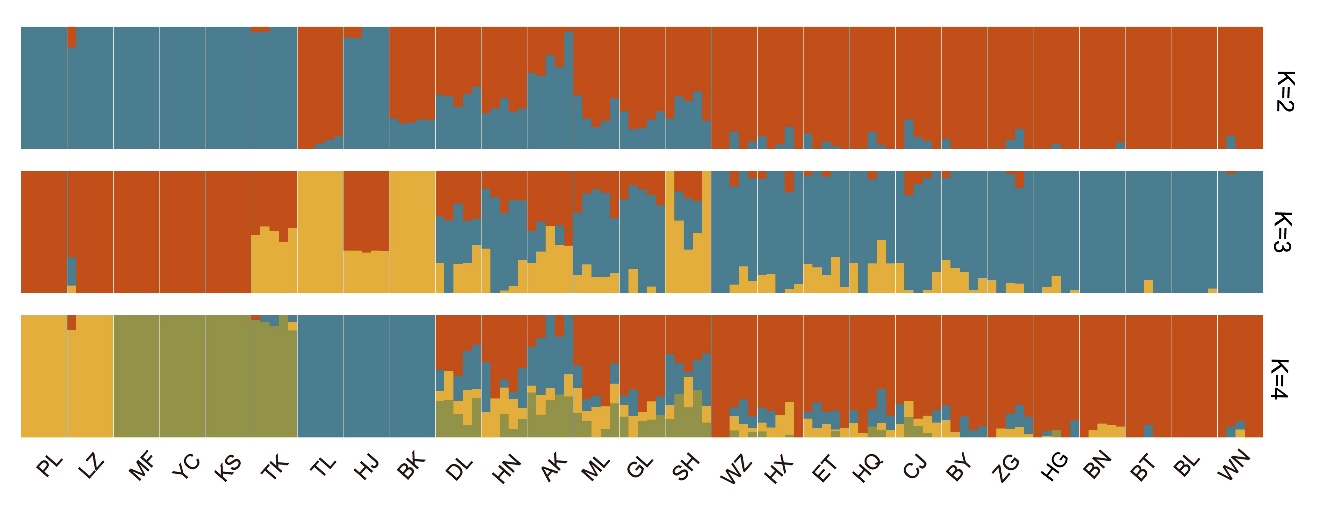


**Figure S1.** **(A)** Cross-validation error for different values of *K* generated by ADMIXTURE. **(B)** ADMIXTURE plot for all *S. breviflora* samples for *K* = 2–4. Each vertical bar represents an individual, with color representing the inferred genomic cluster.


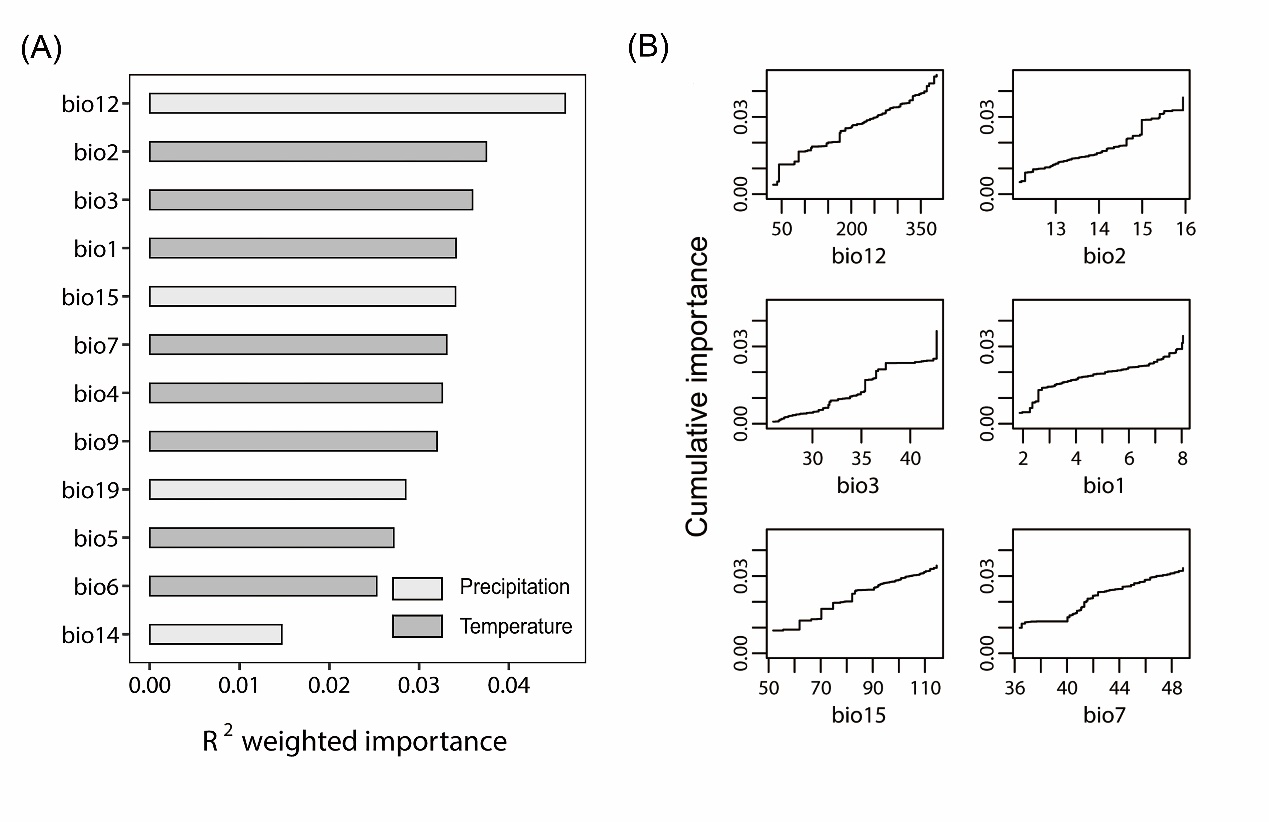


**Figure S2.** The results of gradient forest (GF) analysis of all SNP dataset. **(A)** *R*^2^-weighted importance of environmental variables that explain genetic gradients from GF analysis. **(B)** Cumulative importance of allelic change along the first six environmental gradients. Bio1, annual mean temperature; bio2, mean diurnal range; bio3 isothermality; bio4 temperature seasonality; bio5, max temperature of warmest month; bio6, min temperature of coldest month; bio7, temperature annual range; bio9, mean temperature of driest month; bio12, annual precipitation; bio14, precipitation of driest month; bio15, precipitation seasonality; bio19 precipitation of coldest quarter.


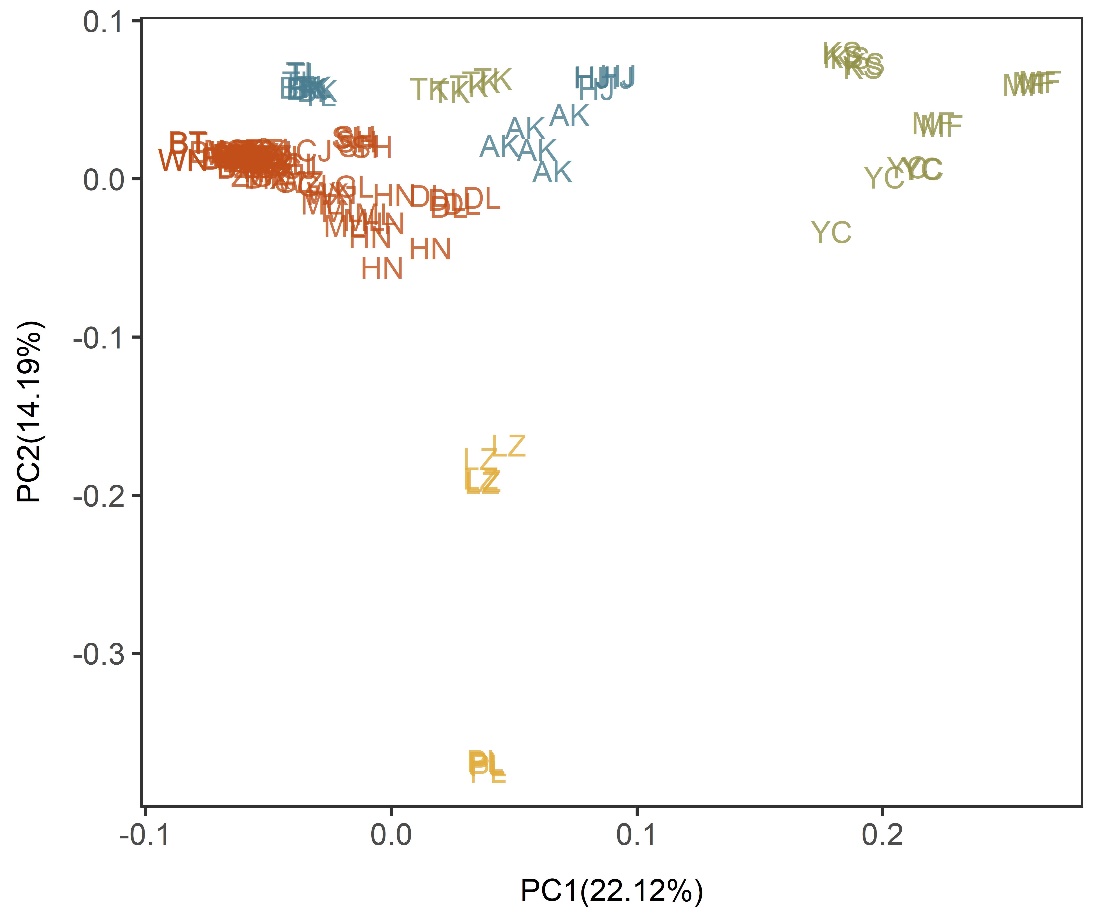


**Figure S3.** Principle component analysis (PCA) based on 25,786 SNPs.
